# Inverse bifurcation analysis: application to simple gene systems

**DOI:** 10.1186/1748-7188-1-11

**Published:** 2006-07-21

**Authors:** James Lu, Heinz W Engl, Peter Schuster

**Affiliations:** 1 Johann Radon Institute for Computational and Applied Mathematics, Austrian Academy of Sciences, Altenbergerstrasse 69, A-4040 Linz, Austria; 2 Theoretical Biochemistry Group, Institute for Theoretical Chemistry, University of Vienna, Währingerstrasse 17, A-1090 Vienna, Austria

## Abstract

**Background:**

Bifurcation analysis has proven to be a powerful method for understanding the qualitative behavior of gene regulatory networks. In addition to the more traditional *forward problem *of determining the mapping from parameter space to the space of model behavior, the *inverse problem *of determining model parameters to result in certain desired properties of the bifurcation diagram provides an attractive methodology for addressing important biological problems. These include understanding how the robustness of qualitative behavior arises from system design as well as providing a way to engineer biological networks with qualitative properties.

**Results:**

We demonstrate that certain inverse bifurcation problems of biological interest may be cast as optimization problems involving minimal distances of reference parameter sets to bifurcation manifolds. This formulation allows for an iterative solution procedure based on performing a sequence of eigen-system computations and one-parameter continuations of solutions, the latter being a standard capability in existing numerical bifurcation software. As applications of the proposed method, we show that the problem of maximizing regions of a given qualitative behavior as well as the reverse engineering of bistable gene switches can be modelled and efficiently solved.

## 1 Background

The use of mathematical models provides tools for the analysis of complex molecular interactions aiming at an understanding of processes occurring in living cells. For many problems in cellular control, stochastic effects and time-delays can be ignored and systems of first-order ordinary differential equations (ODEs) can adequately model the underlying processes. Denoting by *x *and *p *the biochemical concentrations and parameters, respectively, the instantaneous change in *x *is described by the vector field *f*:

 = *f*(*x*, *p*).     (1)

In the study of such systems, an important goal is to understand how the observed physiological behavior arises out of gene network topology and parameters *p*. Some of these questions may be studied via examining the *bifurcation manifolds *Σ of the ODE system, which partition the parameter space into regions of different qualitative behavior (see e.g., [1] for a general overview to bifurcation theory). From ODE models and measured parameters, the *forward problem *of computing the bifurcation diagram has contributed significantly towards elucidating the complex mechanisms underlying cellular processes. For instance, mathematical and symbolic bifurcation analysis has led to an understanding of the possible dynamical behaviors that may arise out of simple gene systems (for a monograph, see [2], examples of more recent papers dealing with natural, designed, and model systems are [3-7]). For cell cycle models, bifurcation diagrams have given biologists a systems-level perspective of the roles played by the various constituent modules, as well as providing the ability to predict the behavior of mutant cells [8,9]. The desire to locate regions in parameter space exhibiting interesting dynamics has led to the development of computational tools for the automatic discovery of bifurcation sets [10].

In contrast, *inverse problems *have only recently attracted attention in biology as a way to unravel the workings of cellular mechanisms. In inverse problems one looks for causes for observed or desired effects [11]. Mathematically, such problems are typically ill-posed, in particular unstable; special mathematical techniques, called "regularization methods", have to be used to cope with this ill-posedness. Many variational and iterative regularization techniques have been developed over the years and applied to a variety of problems in science, engineering and finance (see [11] and some references quoted there, and [12]).

In the current context of cell biology, one would like to address problems such as: which parameter configurations lead to an observed qualitative behaviour of the system ("identification")? How can one introduce a certain qualitative behaviour into the system via parameter variations ("design")? We summarize such problems under the name of *inverse bifurcation problems*, where the task is to map the space of bifurcation diagrams back to the space of parameters. In particular, we consider inverse bifurcation problems of two types: *identification *and *design*. For the former, one would like to infer parameter values from observed bifurcation diagrams and hence the issue of uniqueness is typically of concern. For the latter, one is interested in parameter values that produce the desired outcome, hence uniqueness is not an issue. The notion of "inverse bifurcation" was first introduced into biology in [13]; more recently, another inverse bifurcation problem from mathematical ecology was studied in [14] by integral equation methods. These papers are concerned with the existence and uniqueness of nonlinear terms in equations realizing prescribed bifurcation diagrams and use analytical methods. In contrast, given the size and complexity of gene networks, we take a computational approach in addressing problems of inverse bifurcation. In this paper, we consider problems of moderate size for which the ill-posedness is not yet a crucial issue. Since ill-posedness increases with dimension, a major issue for larger problems will be to use appropriate regularization techniques.

For the *design *type of inverse bifurcation problems, there exists previous work in the engineering literature: The distance to bifurcation manifolds has been introduced to quantify the "parametric robustness" of system designs in [15]; in the context of design of chemical processes, optimization problems with constraints involving distance to bifurcation manifolds have been treated in [16]. For a recent review see [17]. For biological applications as we have them in mind, this issue of parametric robustness is also important. In addition, other geometric properties of the bifurcation diagram are of interest. These include the size of the parameter region resulting in bistability of solutions and the parametric distance between regions of different qualitative behavior. We will develop methodolgies by which inverse bifurcation problems involving the optimization of such quantities can be solved.

In this paper, we show the applicability of our inverse bifurcation algorithms to low dimensional gene systems. We formulate the inverse problems as constrained optimization problems, whose objective function and constraints involve geometric properties of bifurcation diagrams. We demonstrate that these problems can be solved efficiently by applying gradient-based nonlinear optimization algorithms in combination with one-parameter continuation methods to locate bifurcation points. The latter is a standard capability provided by existing bifurcation analysis software (see [1] for references to state-of-the-art numerical implementations).

## 2 Inverse bifurcation analysis

The ODE model (1) defines a mathematical relationship between the parameters *p *and the time course of biochemical concentrations, *x*(*t*). Of the set of all parameters, some describe the biochemical mechanisms constituting the machinery of the regulatory system, while others are parameters to whose variation the gene system should respond. In this paper, we refer to the former as the *system parameters *and the latter as the *control inputs*.

In situations where the qualitative behavior of the regulatory system changes in response to shifts in the control input, the corresponding bifurcation diagram is of importance. In particular, the proper working of the regulatory system may depend on the locations, shapes and sizes of the various regions of qualitative behavior. For instance, in the cell cycle model [8] the correct spatial relationship (in parameter space) of the transitions points is crucial for ensuring the survival of cells. In fact, the model predicts that "dynamically challenged" mutants with shifted bifurcation diagrams may suffer irrecoverably. The resulting effects include difficulties exiting mitosis and decreases in cell mass after each cell division. For this particular model, the inverse bifurcation problem is to map geometric as well as topological relationships in the bifurcation diagrams to conditions on the parameters. Another application of inverse bifurcation in the cell cycle model is for the so-called "Pinocchio effect" involving check points. This effect occur when the necessary conditions for safe progression to the next cell phase are not satisfied hence the regulatory system should delay the state transition as far as possible. Here, the inverse bifurcation problem is to find out how the system may be constructed so that, when triggered by the presence or absence of certain chemicals, the range of bistability is maximized.

In the case of system parameters, they are either regulated by other control mechanisms in the cell, or the behavior of the system is constructed to be insensitive to variation in these parameters [18]. Within an operating region in the control input space, the minimal distance in the system parameter space to bifurcation manifolds provides a quantitative measure of the robustness of the system to environmental perturbations. Here, the inverse bifurcation problem would involve finding the parameter combinations so that the system bifurcation diagram is insensitive to the unregulated parameters. Biological applications of this class of problems include homeostasis and rhythmic pacemakers. Again, the problem of interest is to map some shape property of the bifurcation diagram to conditions on the parameter space.

For inverse bifurcation problems of biological interest such as those described above, the question arises as to how to formulate them mathematically so that the solution can be obtained in a computationally tractable and stable manner. Typically, in biological applications the parameter space is of high dimension. Furthermore, in carrying out inverse analysis, the (forward) bifurcation analysis usually has to be applied a large number of times. In most cases, the former condition precludes formulations based on the use of multi-parameter continuation techniques [19]. Instead, formulations based on continuation along rays in parameter space are preferred. Below, we formulate distance to bifurcation manifolds (first introduced in [15]) in terms of a forward operator and an *l*_2 _functional. Subsequently, we describe the sensitivity of the minimal distance with respect to parameters by adjoint methods.

Consider the splitting of *m*-dimensional parameter space *P *⊂ ℝ^*m *^into input and system parameters, *p *= (*p*_*i*_, *p*_*s*_) ∈ *P*_*i *_× *p*_*s *_. For an ODE system, let Σ denote a bifurcation manifold of interest, consisting of sets in parameter space *P *for which structural stability breaks down [1]. For a given system parameter *p*_*s*_, we further define Σ(*p*_*s*_) ≡ Σ ∩ {*p*_*s*_} as the intersection of Σ with the *p*_*s*_-plane. In Figure 2, the geometric relationship between Σ and Σ(*p*_*s*_) is illustrated. Let the *forward operator F *:*P *→ *P *be a mapping in parameter space, taking a given point to its orthogonal projection on Σ(*p*_*s*_), assumed to be well-defined. That is,

**Figure 1:**
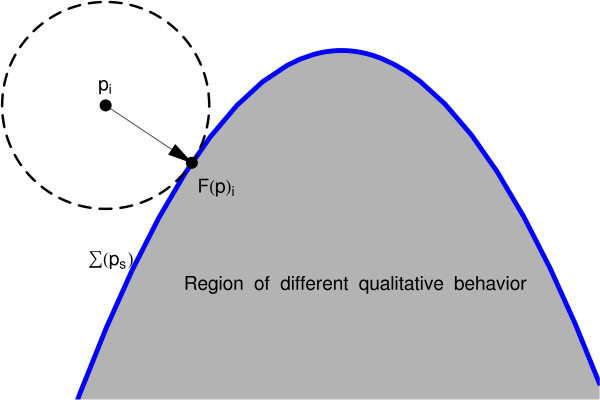
Forward map in the input plane, *P*_*i*_.

**Figure 2:**
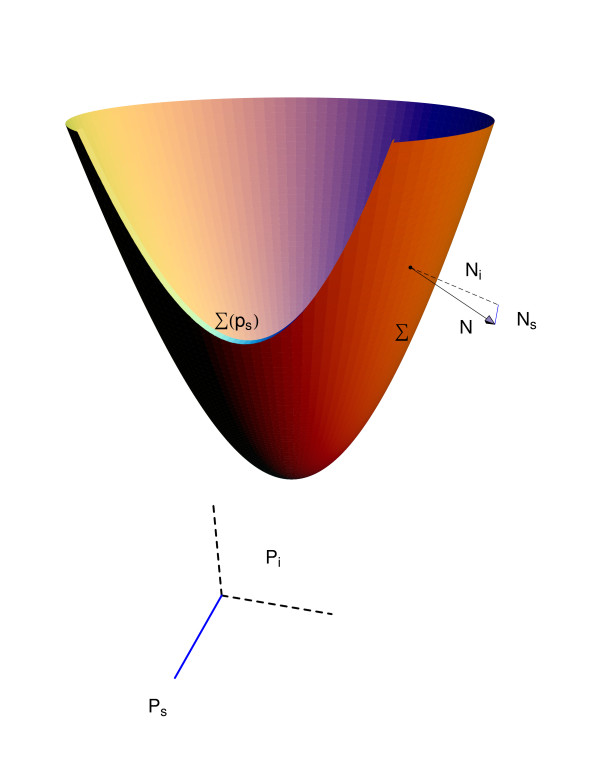
Illustration of normal vector *N *and components *N*_*i*_, *N*_*s*_.

*F*(*p*) ≡ (*F*(*p*)_*i*_, *F*(*p*)_*s*_)



The notation is illustrated in Figure 1.

Using *F*(*p*), the inverse bifurcation problems examined in this paper are mathematically formulated as:



Subject to: *p*_low _≤ *p *≤ *p*_upp_

0 ≤ *c*(*F*(*p*)_*i*_),     (2)

where ||·|| denotes the *l*_2_-norm and *c *: *P*_*i *_→ ℝ^*k *^represents *k*-dimensional nonlinear constraints. In the rest of this section, we discuss adjoint sensitivity analysis of *F*(*p*), which is important for applying gradient-based optimization methods for solving (2) as well as for computing *F*(*p*) iteratively. Denote the linearization of the above map at *p *as *F'*(*p*). The *adjoint operator F*'*(*p*) is defined to satisfy the following: for all *δp*, *δ* ∈ ℝ^*m*^,

<*F*'(*p*)*δp*, *δ*> = <*δp*, *F*'*(*p*)*δ*>,     (3)

where the notation <·, ·> denotes the *l*_2_-inner product. Suppose <·, *l*> is a linearized functional of interest on *P*. Given a parameter perturbation *δp*, the forward functional sensitivity is then given by <*F'*(*p*)*δp*, *l*>. The same sensitivity can be obtained via the *adjoint solution*, *ψ *≡ *F*'*(*p*)*l*. From the definition of the adjoint operator (3), it follows that:

<*F*'(*p*)*δp*, *l*> = <*δp*, *ψ*>.     (4)

That is, for all *δp *∈ *P *the functional sensitivities can be computed via a single linear solution for *ψ *rather than repeated application of the linearized forward operator, *F*'(*p*)*δp*.

For the case where the functional of interest is the *l*_2_-distance *J*(*p*) = ||*F*(*p*)_*i *_- *p*_*i*_||, the adjoint solution for the linearization *J*'(*p*)(·) is given in terms of the vector normal to the manifold Σ. Denoting *N*_*i *_and *N*_*s *_as the components of the normal vector in *P*_*i *_and *P*_*s *_respectively, it can be shown that (up to sign),



To fix the sign of *ψ*, the component *N*_*i *_is chosen to be *p*_*i *_- *F*(*p*)_*i*_. Figure 2 illustrates the components of the vector *N *normal to the manifold Σ.

Thus, obtaining expressions for the adjoint vector for various bifurcations of interest reduces to the problem of deriving the associated normal vectors, *N*_*s*_. Under certain transversality conditions, the normal vectors for several codimension-one and higher bifurcations have been derived [16,17]. Here we consider the generic codimension-one bifurcations, namely saddle-node and Hopf bifurcations. Let the left and right critical eigenvectors of *f*_*x *_at the given bifurcation point be denoted by *w *and *v *respectively. That is, *w *and *v *solve the following eigen-systems for the critical eigenvalue *ω*_crit _and its conjugate _crit_,

*f*_*x*_*v *= *ω*_crit _*v*

.

**Figure 3:**
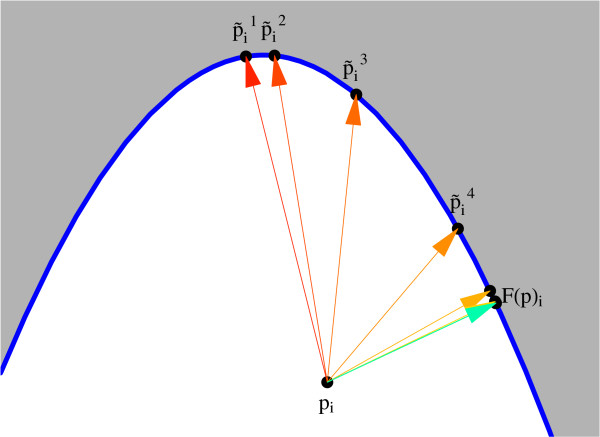
Demonstration of iterative procedure for computing *F*(*p*).

The expressions for normal vectors are given as:



where superscript *H *denotes conjugate transpose and . These expressions above prescribe the components of the adjoint solution, thus enabling efficient gradient calculation via (4). Now, we briefly mention methods for computing the projection *F*(*p*). Methods of iterative and direct type for finding the (locally) closest bifurcation point have been derived [17]. In the current work we use the former approach, based on using the component of the normal vector in the input plane, *N*_*i*_. Provided certain conditions on the principal curvatures of Σ(*p*_*s*_) are met, geometric convergence is assured. The algorithm is discussed in Section 3. Figure 3 illustrates the method in a simple example, producing a sequence of iterates (_*i*_) converging to the point *F*(*p*)_*i *_that is closest to a (non-convex) neighboring region with respect to *p*_*i*_.

Finally, we mention that the minimal distance functional can be used to model many other problems of interest. For instance, it can serve as an estimate of the separation between a reference manifold Σ_ref _and a given region of qualitative behavior, or the size of the region of a qualitative behavior via the maximum radius of the inscribed sphere.

## 3 Algorithm and software implementation

**Figure 4:**
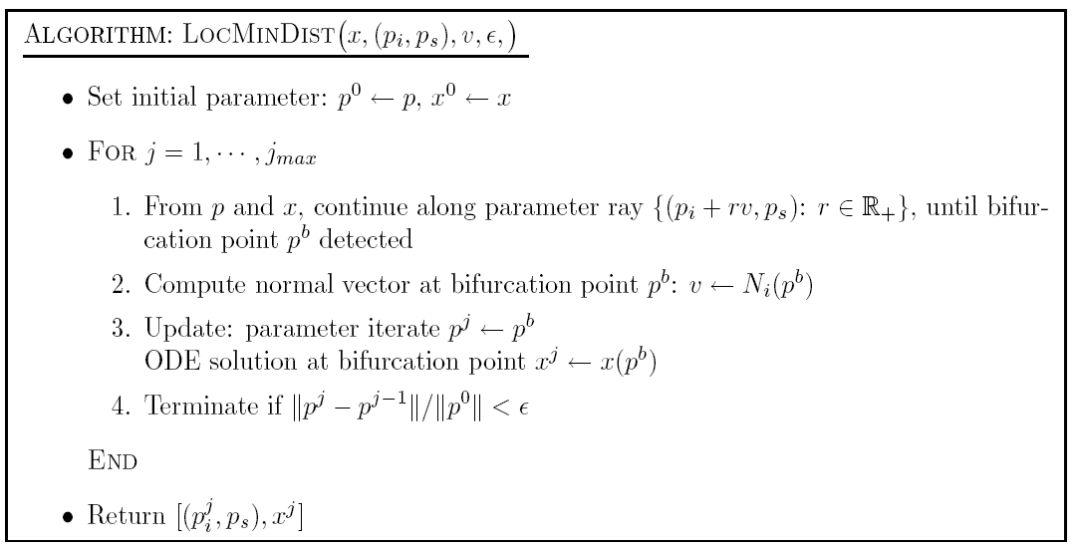
Algorithm APPLYF.

**Figure 5:**
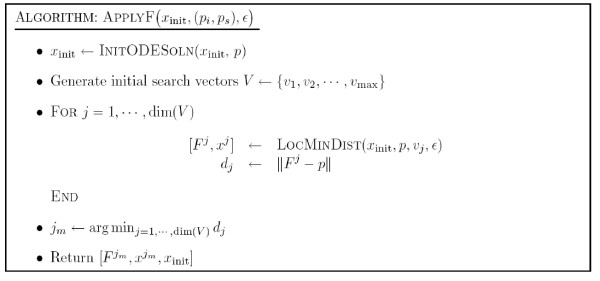
Algorithm LOCMlNDlST.

**Figure 6:**
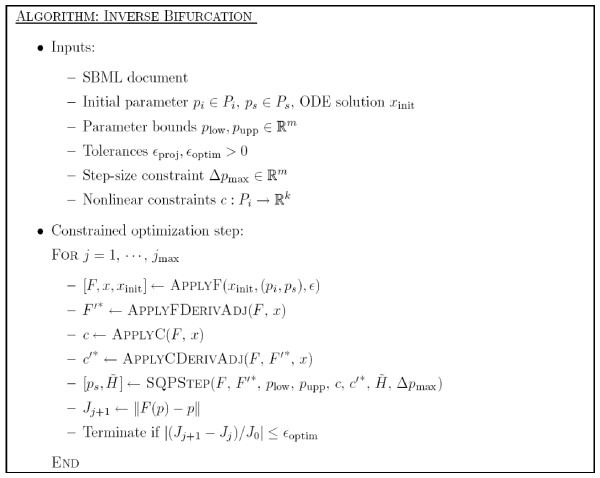
Algorithm INVERSE BIFURCATION.

Here we give an outline of the algorithm for general inverse bifurcation problems. The main ingredients are applications of the projection operator *F*(*p*), as well as the adjoint, *F*'*(*p*). The computation of the former is denoted by the routine APPLYF (see Figure 4). Each time APPLYF is called, corrector steps (using, for instance, Newton's method) have to be carried out on the previously computed *x*_init _to find the initial solution for the current value of *p*_*s*_. Once the corrected solution *x*_corr _is computed, the iterative procedure LOCMINDIST (see Figure 5) is called to compute the nearest point on the bifurcation manifold of interest. This procedure is based on a series of one-parameter continuations and gradient calculations according to (6). The inputs include the following: *v *the initial search direction in parameter space; *ε*_proj _the relative tolerance on the iterative proceure. In general, several search directions (denoted by *V *= {*v*_1_,⋯, *v*_max_}) have to be used to approximate the globally closest point. However, for the examples shown in the paper the initial search space *V *is only 1 dimensional. Once *F*(*p*) and the corresponding solution are obtained, *F*'*(*p*) is then computed. The derivative information, together with constraints (*p*_low_, *p*_upp_, c(*p*)) and Hessian approximation (HessA) are then used to compute an SQP step and update the Hessian. Figure 6 describes the inverse bifurcation algorithm.

Our Inverse Bifurcation Toolbox is an implementation of the above described algorithm. It combines the capability of several packages: the MATCONT package [20] for performing the underlying one-parameter continuations, the MATLAB Optimization Toolbox [21] for performing gradient-based constrained optimization, the MathSBML package [22] for reading in biological models in the SBML format and finally the Mathematica Symbolic Toolbox for MATLAB [23] for communications. The combination of MATLAB and Mathematica has the advantage of allowing building on existing freely-available software.

After the SBML model is read in via MathSBML, the vector fields are differentiated symbolically to obtain expressions for *f*_*x*_*, f*_*p*_*, f*_*xp *_and *f*_*xx*_. Subsequently, they are numerically evaluated when called by MATCONT. Reparametrization of parameters is done automatically to allow continuation in arbitrary directions. Presently, the toolbox is able to handle bifurcations of saddle-node and Hopf type. Augmentation to include bifurcations of limit cycles is currently underway.

Given the limitations of the current implementation, our software is suitable for handling problems involving tens of variables and parameters but possibly not suited for problem dimensionality on the order of hundreds or higher. Further algorithm development is needed to significantly upscale the method. In addition, in high dimensional applications instabilities may appear and hence appropriate regularization techniques need to be developed (e.g., stopping rules in the case of iterative methods).

## 4 Applications

### 4.1 Finding distance to oscillation in a 4 gene model

To understand design principles underlying biological systems, simple oscillatory and switch-like systems have been constructed experimentally. Several systems based on *E. coli *[24,25] have been successfully demonstrated. Recently, components of the Lac and Ntr systems have been used to construct a genetic clock that upon change in connectivity, exhibits switch behavior [26]. After ignoring the dynamics of the read-out gene, the system of three equations for the concentrations of mRNA (*x*_1_, *x*_3_, *x*_5_) and proteins (*x*_2_, *x*_4_, *x*_6_) is given as,



where *β*_*i *_are rate constants. The rates of transcription are represented by the following tri-phasic functions,



where *g*_*jk *_are kinetic-order parameters, typically less than 4.

Given a stable, steady solution for a gene system with a set of nominal parameters, a relevant engineering question is how to construct a genetic oscillator. For the current system, the analytical solutions for the bifurcation manifolds are available via the Routh-Hurwitz stability criterion ([26], supplementary data). However, such analysis cannot be easily carried out for more complex models. Instead, computational methods have to be used.

**Figure 7:**
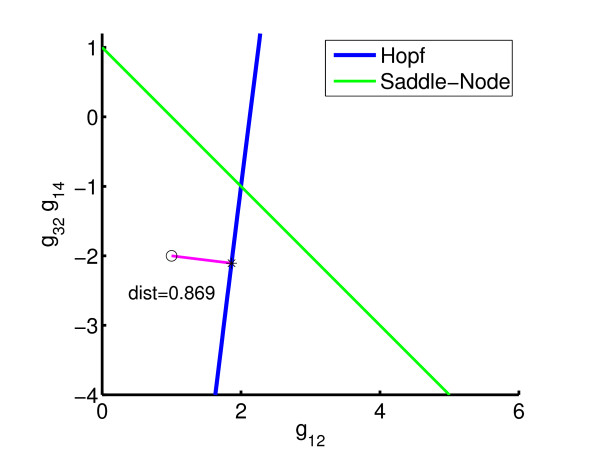
Initial bifurcation diagram for 4 gene model: *β*_1 _= *β*_3 _= 222, *β*_2 _= *β*_4 _= 1.39.

Suppose the stable system has rate parameter *β*_1 _= *β*_3 _= 22.2, *β*_2 _= *β*_4 _= 1.39, kinetic-order parameters g_32 _= -2, *g*_12 _= *g*_14 _= *g*_54 _= 1. If in practice only the rate parameters *β*_*i *_can be varied, the question is how best to construct an oscillatory system. The bifurcation diagram for the model is shown in Figure 7, where it can be seen that for the nominal values of *g*_*jk *_the system is stable and far away from the line of Hopf bifurcation. The inverse bifurcation question is: do there exist parameters *β*_*i *_such that the system is oscillatory? We consider the problem with the following parameter constraints:

0.1 ≤ *β*_1_, *β*_3 _≤ 25

0.1 ≤ *β*_2_, *β*_4 _≤ 4.

**Figure 8:**
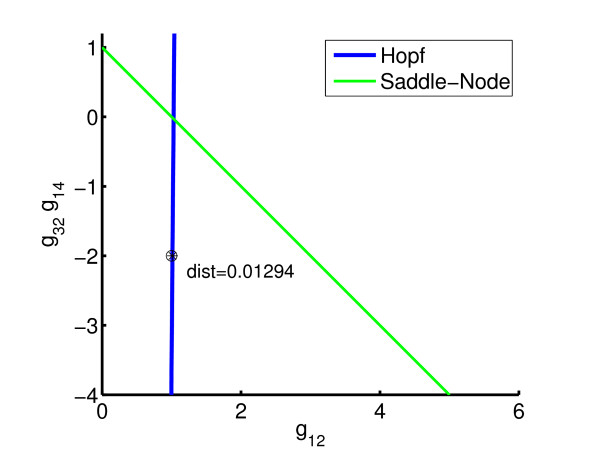
Optimized bifurcation diagram for 4 gene model: *β*_1 _= 22.186, *β*_2 _= 4, *β*_3 _= 22.145, *β*_4 _= 0.1.

By minimizing the distance from the nominal parameter point (*g*_12 _= 1, g_32_*g*_14 _= -2) to the Hopf bifurcation line, the system can be made to lie on the boundary of the stable regime; see Figure 8. Table 1 shows the iteration counts required to obtain the result, using Sequential Quadratic Programming (SQP) with line-search [27]. Owing to the use of line-search, each optimization iteration entails a number of functional evaluations, this in turn requiring a number of one-parameter continuation iterations to find the (locally) closest point on Σ(*p*_*s*_). The optimality tolerance on the objective is set as 10^-3 ^relative to its initial value and the relative parameter tolerance for approximating *F*(*p*) is set to 10^-4^. Clearly, the number of one-parameter continuations is significantly higher than the optimization iterations. Thus, for high dimensional examples it is important that the former can be carried out efficiently.

**Table 1:** Iteration summary

	Optim. iter.	Func. eval.	One-param contin.
4 gene system	7	15	60

### 4.2 Maximizing regime of oscillations in repressilator system

Motivated by biological applications such as increasing the robustness of rhythmic pacemakers, we consider maximizing the region of oscillations with respect to a given operating point. We utilize a case of the generalized repressilator [7] as our test system.

**Figure 9:**
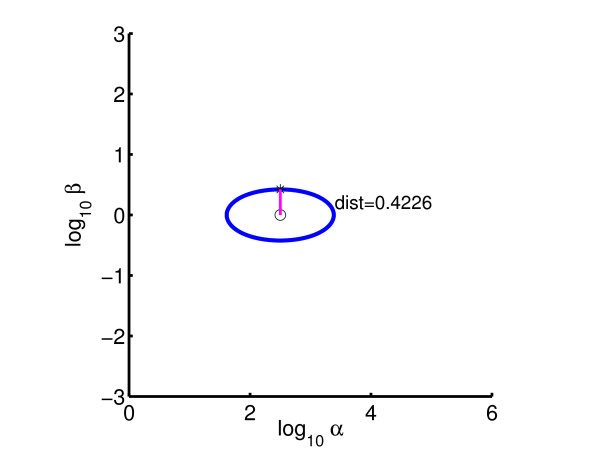
Initial bifurcation diagram for symmetric repressilator: *δ *= 10^-3^, *h *= 1.5.

**Figure 10:**
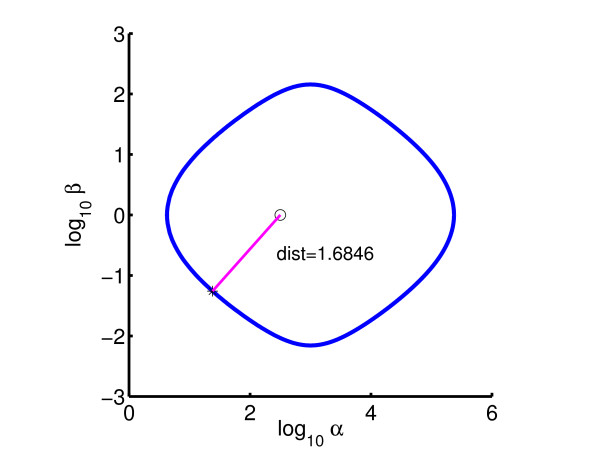
Optimized bifurcation diagram for symmetric repressilator: *δ *= 10^-4^, *h *= 2.

After non-dimensionalizing, an ODE system for the protein and RNA concentrations *x*_*i *_and *y*_*i *_is obtained, with the following dimensionless parameters: *α*_*i*_, *β*_*i *_are the ratios of degradation rates, *δ*_*i *_represents the leakiness of the gene transcription, *h*_*i *_the Hill-coefficient reflecting the degree of cooperativity of gene transcription,



In this example, we take *n *= 3 and examine the symmetric case, i.e., *α*_*i *_= *α*, *β *= *β*, *etc*. Analysis shows that for ranges of parameters *α *and *β*, the system can exhibit oscillations or be in a stable, steady-state [7]. Thus, we take *p*_*i *_= (*α*, *β*) as the input parameters and seek values of *p*_*s *_= (*δ*, *h*) within the constraint set,

(10^-4^, 0) ≤ (*δ*, *h*) ≤ (10^-1^, 2)

so as to maximize the minimal distance to a Hopf bifurcation with respect to a reference point (*α *= 10^2.5^, *β *= 10^0^). Figure 9 shows the initial bifurcation diagram and the computed minimal distance from the reference point. The result of the inverse bifurcation analysis shows that the optimum solution is found at (*δ*, *h*) = (10^-4^, 2); thus, the inequality constraints are active equality constraints. Moreover, the signs of the Lagrange multipliers for the inequality constraints show that the (local) optimum distance to the Hopf bifurcation manifold increases if the upper bound *h*_upp _is increased and/or the lower bound *δ*_low _is decreased. Figure 10 shows the bifurcation diagram with the optimized parameters.

Table 2 shows the iteration counts required to obtain the result. The tolerances for optimization are the same as those of Section 4.1.

**Table 2:** Iteration summary

	Optim. iter.	Func. eval.	One-param contin.
Symmetric repressilator	2	5	48

### 4.3 Engineering bistable switch in *G*_1_/*S *transition

We consider the reverse engineering of check points in mammalian cell phase transition. In particular, we use a 9 state, 40 parameter differential equation model [28]. In the publication describing the model, parameter scanning is done to find out parameter values that achieve certain behaviors, such as changing the threshold for the onset of synthesis (S) phase from the *G*_1 _state and conditions ensuring irreversibility of the gene switch. Using our inverse bifurcation analysis, specifying a desired behavior on the transition points gives rise to conditions on the parameters in a more systematic and efficient manner.

**Figure 11:**
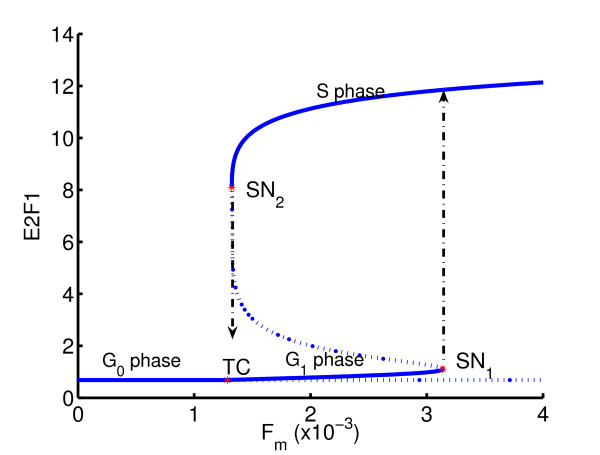
Bifurcation diagram of cell phase transition.

Figure 11 shows the bifurcation diagram for the regulatory module of the mammalian *G*_1_/*S *transition. The bifurcation parameter is the strength of mitogenic stimulation, *F*_*m *_. The quantity of primary interest is the level of E2F1, which is a transcription factor targeting genes underlying cell cycle progression. For sufficiently low level of *F*_*m*_, the level of E2F1 stays at a constant low value. At a critical value of *F*_*m *_a transcritical point is reached, above which the level of E2F1 starts to increase with *F*_*m *_. As the level of *F*_*m *_increases further, a saddle-node (SN_1_) is reached and the level of E2F1 undergoes a discontinuous jump upwards, thereby triggering the S phase of the cell cycle.

#### 4.3.1 Example 1: engineer irreversibility

First, we mention a number of main species and their interactions. The regulatory system consists of a core double inhibitor-activator module as well as several positive-feedback loops. The transcription factor E2F1 activates itself as well as pRB, a tumor suppressor. In turn, pRB inhibits itself as well as E2F1. The following equations describe this core module:



Another important collection of species is AP-1, which is used to denote a family of transcription factors that mediate the mitogenic signal *F*_*m *_. The family AP-1 is also activated by E2F1, with feedback strength *k*_25_. The dynamics for the concentration of the family [AP-1] is modelled as:



In the first example, we consider the construction of an irreversible gene switch via parameter changes. It has been demonstrated [28] that for some value of *k*_25 _in (8), the *G*_1_/*S *transition becomes irreversible. That is, if *F*_*m *_is increased beyond a certain point, a subsequent decrease to zero will not lead to a *G*_0 _state with a low level of E2F1.

**Figure 12:**
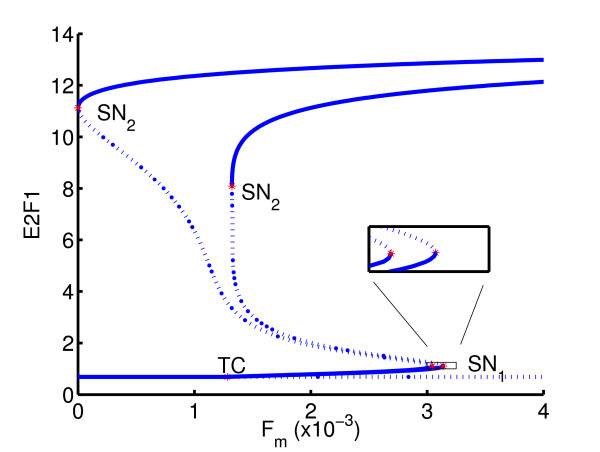
Example 1: initial and optimized gene switches.

Geometrically, the inverse problem is to find the value of *k*_25 _such that the *x*-abscissa of the upper saddle node (SN_2_) is as close to zero as possible. As inequality constraints, we take 0.1 ≤ *k*_25 _≤ 1.5. The result of the inverse analysis shows that the feedback strength should increase from its initial value of *k*_25 _= 0.9 to *k*_25 _= 1.099, resulting in a change of bifurcation diagram shown in Figure 12. Row 1 of Table 3 shows the number of optimization iterations as well as the number of functional evaluations (which in this case equals the number of one-parameter continuations) required to reach a tolerance of 10^-3 ^on the function value.

**Table 3:** Iteration summary

	Optim. iter.	Func. eval.
Example 1: irreversibility	6	14
Example 2: irreversibility, fixed *G*_1_/*S*	9	22

#### 4.3.2 Example 2: engineer irreversibility, fixed *G*_1_/*S *transition

In this example, we consider an extension of the previous problem. Here, we would like to engineer an irreversible switch with the additional constraint that the *x*-abscissa of the *G*_1_/*S *transition point SN_1 _should be fixed at the value of the nominal system. In addition to allowing 0.1 ≤ *k*_25 _≤ 1.5 as before, we also modify the stability of E2F1 and AP-1. That is, we relax the values of *φ*_E2F1 _and *φ*_AP-1 _in (7) and (8) from from their fixed nominal values 0.1 and 0.01 respectively, to allow searching within the ranges,

0.08 ≤ *φ*_E2F1 _≤ 0.12

0.008 ≤ *φ*_AP-1 _≤ 0.012.

**Figure 13:**
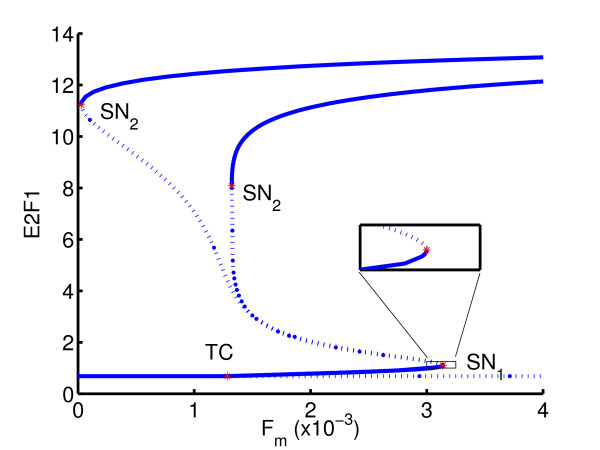
Example 2: initial and optimized gene switches.

Mathematically, the inverse problem is formulated as an nonlinear optimization problem with equality constraint on the *x*-abscissa of the *G*_1_/*S *point, SN_1_. The bifurcation diagram corresponding to the solution (*k*_25 _= 1.182, *φ*_E2F1 _= 0.0992, *φ*_AP-1 _= 0.0109) is given in Figure 13. Row 2 of Table 3 summarizes the number of iterations required.

## 5 Conclusion

We propose an inverse problems methodology to address a class of problems arising in the study and design of gene systems. The methodology is based on the (inverse) map from the space of bifurcation diagrams to the space of biochemical parameters. Within this general inverse bifurcation framework, we show that many questions of biological interest may be formulated as optimization problems involving minimal distances to bifurcation manifolds. Adjoint sensitivity analysis is carried out to allow for efficient solution using gradient-based optimization methods.

Several general questions remain for the methodology: what biological problems can be formulated in terms of geometric properties of the bifurcation diagrams? Given desired geometric properties for bifurcation diagrams, how best can the problem be formulated mathematically to allow for tractable solution? Finally, we remark that the development of appropriate numerical methods, novel computational strategies and regularization techniques are necessary to allow for the upscaling of the current computational tool to high dimensional biological applications.
